# Identifying connectivity for two sympatric carnivores in human-dominated landscapes in central Iran

**DOI:** 10.1371/journal.pone.0269179

**Published:** 2022-06-16

**Authors:** Sahar Rezaei, Alireza Mohammadi, Roberta Bencini, Thomas Rooney, Morteza Naderi

**Affiliations:** 1 Faculty of Science Engineering, Department of Biological Sciences, University of Arkansas, Fayetteville, Arkansas, United States of America; 2 Faculty of Natural Resources, Department of Environmental Science and Engineering, University of Jiroft, Jiroft, Iran; 3 Department of Agriculture and Environment, University of Western Australia UWA, Perth, Australia; 4 Department of Biological Science, Wright State University, Dayton, Ohio, United States of America; 5 Department of Agriculture and Environment, University of Arak, Arak, Iran; Amity University, INDIA

## Abstract

Central Iran supports a diversity of carnivores, most of which are threatened by habitat loss and fragmentation. Carnivore conservation requires the identification and preservation of core habitats and ensuring connectivity between them. In the present study, we used species distribution modeling to predict habitat suitability and connectivity modeling to predict linkage (resistant kernel and factorial least-cost path analyses) for grey wolf and golden jackal in central Iran. For grey wolf, elevation, topographic ruggedness, and distance to Conservation Areas (CAs) were the strongest predictors; for golden jackal, distance to human settlements, dump sites and topographic ruggedness were the most influential variables in predicting the occurrence of this species. Our results also indicated a high potential for large parts of the landscape to support the occurrence of these two canid species. The largest and the most crucial core habitats and corridor paths for the conservation of both species are located in the southern part of the study landscape. We found a small overlap between golden jackal corridor paths and core habitats with CAs, which has important implications for conservation and future viability of the golden jackal populations. Some sections of core areas are bisected by roads, where most vehicle collisions with grey wolf and golden jackal occurred. To minimize mortality risk, we propose that successful conservation of both species will necessitate integrated landscape-level management, as well as conservation of core areas and corridors and development of mitigation strategies to reduce vehicle collisions.

## 1. Introduction

Habitat loss and fragmentation can have a negative influence on ecosystems and species by diminishing habitat carrying capacity and raising mortality risk, as well as inhibiting individual dispersal and consequently gene dissemination across landscapes [1]. This synergistically increases the risk of local extinction [2,3]. Large carnivores are particularly vulnerable to habitat fragmentation and habitat loss [4]. They live in low densities and typically have large home ranges [5,6]. Carnivores’ large area requirements result in their need for vast and connected habitat areas where they are protected from anthropogenic disturbance. Carnivore species often been affected by increasing land use change and habitat fragmentation, which has reduced habitat areas and increased isolation, leading to a synergy of increased direct human-caused mortality, reduced local carrying capacity, and reduced ability for populations to be integrated by dispersal [7].

Moreover, they are more likely than animals with reduced mobility and smaller home ranges to be exposed to road crossings and other high risk places in the landscape [8]. In central Iran, vehicle collisions pose a significant threat to wildlife species [9]. Roads, especially those with high traffic, disrupt both structural and functional connectivity for large carnivores, including grey wolf (*Canis lupus*) and golden jackal *(Canis aureus*), and can lead to reduced gene flow among sub-populations [9]. Highway planners need accurate data to determine when and where specific species are vulnerable to high road-kill rates so that mitigation measures can be implemented at the road design and/or discovery stages [10,11].

Large carnivore conservation requires both protection of extensive core areas and the establishment of movement corridors among them [12], particularly when core habitat patches are isolated by road networks [13]. Long-term species conservation depends on connectivity, which is essential for preserving the genetic and demographic processes that ensure long-term viability [14]. Connectivity of populations is of paramount importance to both conserve species locally and to secure their range shifts in response to future hazards such as land use change [15,16] and climate change [17–19]. Enhancing conservation network connectivity can help to mitigate the negative effects of habitat loss and fragmentation [20,21].

Connectivity models provide practical tools for assessing potential fragmentation effects of roads on wildlife and help inform management and conservation planning [22]. For connectivity analysis, a number of methods have been suggested, including least-cost path modeling [23], current flow [24], factorial least-cost path density [25], resistant kernels [26] and randomized shortest path algorithm [27]. The factorial least-cost path and cumulative resistant kernel approaches are particularly useful when employed in combination to accurately identify core habitats, fracture zones and corridors across a broad landscape [12,28,29], with the particular advantage that they both allow the incorporation of biologically realistic dispersal abilities into the predictions, which has been shown to dominate connectivity estimation [30].

Understanding the different factors that have an impact on species distribution and habitat selection is important for carnivore conservation and management [2,9,31]. Different habitat suitability models are available for investigating species distributions. Machine learning models (e.g., Random Forest) have advantages over standard regression models as they are able to capture nonlinear patterns as well as linear correlation. Also, in contrast to machine learning models, the validity of regression models depends on several assumptions such as normality, data independence, and additivity which are rarely valid in ecological context [32,33].

Ensemble modeling, in which several species distribution models (SDMs) are combined to quantify a range of predictions across more than one set of uncertainty sources, has been found to increase the accuracy of model predictions [9,34] and decrease the uncertainty associated with using a single SDM [35].

Grey wolf and golden jackal, both are two of the most widely distributed carnivores globally and in Iran [36–41]. Generalist diet and plasticity in habitat selection has enabled these canids to occur across a wide range of habitats including human dominated landscapes [9,41,42] However, following reductions in prey species density in Iranian Conservation Areas (Cas) [43], the occurrence of these species in rural regions, where they may get food from pastoral sources, such as livestock, has increased over the last several decades [44–46]. Consequently, they have become highly vulnerable to conflict with humans [47]. For this reason, identifying connectivity and core habitat among these residual populations could assist in conservation of these and other carnivore species and also while guiding conflict management [48].

Data regarding the ecology of grey wolf in Iran is limited to a few studies on diet and foraging habits [44,49], conflict [50], den site selection [51] and local people’s attitudes [45]. Similarly, there is little information about golden jackal ecology in Iran. Only some studies on diet [52], habitat use [41], and connectivity [9]. Other aspects important for the management of the species, such as core habitats, distribution, and connectivity, are still poorly known [53]. The current knowledge on the ecology of golden jackal and grey wolf in Iran remains limited, as only one study has been devoted to habitat use of each of them: a study on grey wolf habitat suitability in central Iran [9] and research on golden jackal habitat use in Iran [41]. As elsewhere, both species are also under anthropogenic pressure in Iran, suffering direct persecution due to low public acceptance, livestock depredation and human attacks [50,53,54].

In this study, we addressed three main objectives regarding grey wolf and golden jackal distribution and connectivity in central Iran. First, we determined the most important environmental and anthropogenic factors influencing habitat suitability for both species and mapped habitat quality. Second, we defined core areas for each species using resistant kernel modeling [26], and identified corridor routes among these core areas using factorial least-cost path modeling [55]. Third, we used spatial randomization of vehicle collision locations to test the predictive ability of resistant kernel and factorial least-cost path predictions of movement [28]. More specifically, we evaluated this hypothesis that golden jackals are known to frequent villages [9] while grey wolves tend to avoid humans [51]. Therefore, we expected that both species would show a different response to human settlement, with grey wolves avoiding areas near settlements and roads, and selecting higher elevations while golden jackals would show an opposite response.

The results of this research provide clarity on the drivers of habitat quality for both species, and the patterns of habitat extent and connectivity for these species across Central Iran which is critical for management of both species especially outside CAs.

## 2. Materials and methods

### 2.1 Study area

**Fig 1:**
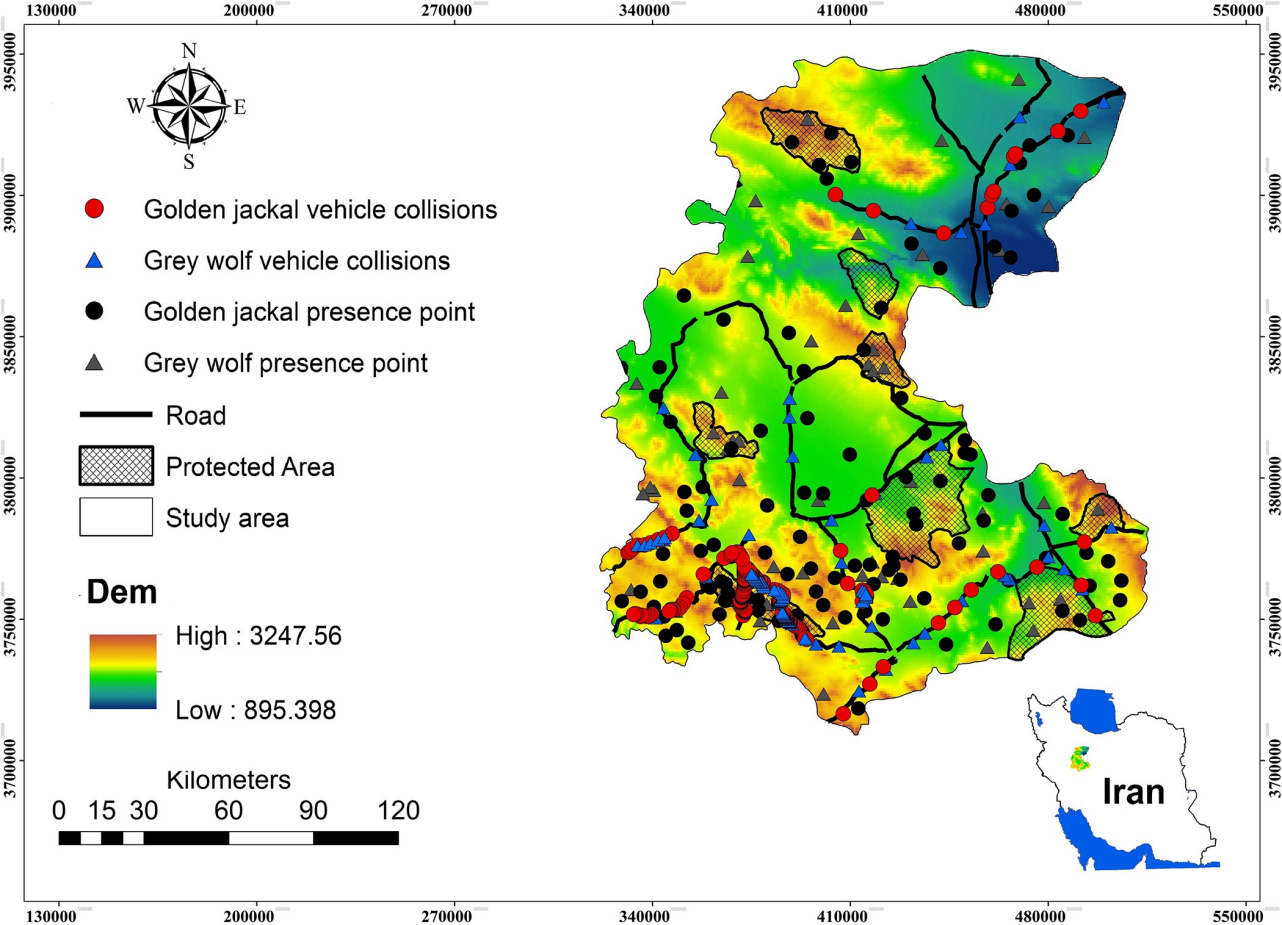
Presence locations and vehicle collisions of the grey wolf and golden jackal in central of Iran (Markazi Province). Dem indicates elevation (m). Contains information from OpenStreetMap and OpenStreetMap Foundation, which is made available under the Open Database License. Republished from [https://markazi.doe.ir/] under a CC BY license, with permission from [Markazi Province Office of Department of Environment (DOE)], original copyright [2021].

The study was conducted across central Iran (33^o^30 to 30^o^535 N; 48^o^57’ to 57^o^51’ E) (Fig 1). This area is bounded between the central desert and the junction of the Alborz and Zagros faults. Despite the arid and semi-arid environmental conditions, this part of Iran supports a high diversity of large and medium-sized carnivores, including grey wolf (*Canis lupus*), golden jackal (*Canis aureus*), red fox (*Vulpes vulpes*), striped hyaena (*Hyaena hyaena*), African wildcat (*Felis lybica*), Persian leopard (*Panthera pardus saxicolor*) and caracal (*Caracal caracal*) [56]. The region also supports three ungulate species (wild sheep (*Ovis orientalis*), goitered gazelle (*Gazella subguturosa*) and wild goat (*Capra aegagrus*) and it includes the vegetation types dominated by *Artemisia* spp., *Scariola orientalis*, *Astragalus* spp. and *Euphorbia* spp. In this landscape there are two wildlife refuges (WRs; IUCN category IV; 2322.01 km^2^), two Conservation Areas (CAs; IUCN category V; 1366.47 km^2^) and five no-hunting areas (NHAs; no IUCN category; 1315.01 km^2^) for protecting biodiversity. CAs are the largest protected areas in terms of both size and number. One-fifth of these lands are entirely protected by agreement (High Council of Environment), while the rest can be used for mining, grazing, and other purposes by the local community. The conservation of animal life is, of course, more important in Wildlife Refuges (WRs). In the core zones of CAs and WRs, livestock grazing is restricted, and ungulate hunting is prohibited in all reserves [57].

### 2.2 Species occurrence data and environmental variables

Occurrence locations of golden jackal and grey wolf between the years 2000 and 2009 were obtained by the first author in collaboration with field surveys and provincial offices of Markazi Department of Environment rangers (S1 Table). During the field visits, all observations including footprints, feces and resting places were regarded as the species presence points. To address the effects of spatial bias due to uneven sampling efforts, we calculated the global Moran’s I test in Arc GIS 10.7 (S1 Fig). The result of the index (S1 Fig) showed that the occurrence points were not spatially correlated. In total, we collected 95 and 113 presence points of grey wolf and golden jackal, respectively.

To predict the distribution and habitat selection of both target species, we chose a suite of environmental factors based on the ecological requirements of grey wolf and golden jackal (S2 Table). The environmental variables were classified into three categories including topography (elevation, slope and topographic roughness), vegetation (vegetation cover, and Normalized Difference Vegetation Index; NDVI). Also, anthropogenic variables were classified into three categories including human disturbance (distance to roads, distance to villages, and distance to dump sites). Moreover, we calculated distance to CAs as prey availability variable.

A digital elevation model (DEM) from the 30m Shuttle Radar Topography Mission (SRTM, downloaded from http://earthexplorer.usgs.gov), was used to calculate slope (using Surface Tool in Spatial Analyst Tools) and surface roughness variables (Geomorphometry and Gradient Metrics toolkit) [58] in ArcGIS 10.2.

To calculate NDVI, we extracted red and near infrared bands of Landsat 8 OLI images for the year 2016 at 30m resolution and calculated the index using the Image Analysis tool in ArcGIS v10.2. For vegetation cover, vegetation types with a density greater than 25% were extracted from the research area’s land cover/land use map (Iranian Forests, Range and Watershed Management Organization, IFRWO) (http://www.frw.ir).

We calculated Euclidean distance to human settlements (https://mapcruzin.com/free-iran-arcgis-maps-shapefiles.htm), roads (https://data.humdata.org/dataset/wfp-geonode-iran-road-network-main-roads), CAs (Markazi Province Office of Department of Environment (DOE)) and dump sites using the Spatial Analyst tool in ArcGIS 10.2. The degree of multicollinearity between the predictors was tested by calculating the Pearson correlation coefficient between pairs of the variables and based on the threshold value of 0.7 [59]. As a result, we only found high collinearity between two variables including slope and topographic roughness.

### 2.3 Habitat modeling

We used an ensemble modeling approach to predict habitat suitability for both species. Ensemble modeling is a powerful approach that combines predictions from different models [34]. The accuracy of the model is increased by fitting several suitability models, the uncertainty associated with using a single model is decreased, and finally, a range of predictions is explored across more than one set of uncertainty sources [34].

Our ensemble models were created by weighted averaging seven different models using the biomod2 R package [60]. Biomod2 was chosen because it is a well-known and well-established software [61]. These models included two regression-based models (Generalized Linear Model [GLM], and Multivariate Adaptive Regression Splines [MARS]) and three machine-learning models (Maximum Entropy [MaxEnt], Random Forest [RF], and Generalized Boosting Model [GBM].

For each species, we evaluated and compared the performance of each habitat suitability model and the ensemble model AUC [62] and we considered a model with AUC > 0.9 as excellent, 0.8–0.9 as good, 0.7–0.8 as moderate and 0.6–0.7 as poor. We took a model with TSS > 0.75 as indicating excellent, 0.4–0.75 as good and < 0.4 as poor [62]. Variables contribution for each model of each species was calculated in Biomod2. The response curves of presence points to the most significant variables in each model were produced and interpreted for each species.

### 2.4 Connectivity analyses

To estimate landscape resistance, we converted the habitat suitability maps to resistance maps using a negative exponential function using this equation:

R=1000^(‐1×HS)

Where R represents the cost resistance value assigned to each pixel and HS represents the predicted habitat suitability derived from the suitability models described above [63,64]. We rescaled the resistance values to a range between 1 and 10 by linear interpolation, such that minimum resistance (Rmin) was 1 when HS was 1, and maximum resistance (Rmax) was 10 when HS was 0 [64].

We used the universal corridor network simulator UNICOR; [65] to create two sets of connectivity predictions including (a) resistant kernels [26] and (b) factorial least-cost paths [25]. The factorial least-cost path analysis done in the UNICOR simulator applies Dijkstra’s algorithm to resolve the single-source shortest path issue from every mapped species occurrence location on a landscape to every other occurrence location [65]. The analysis produces the sum of predicted least-cost paths from each source point to each destination point. The resistant kernel algorithm calculates the cumulative resistance cost-weighted dispersal kernel around each source point up to a user-defined dispersal threshold [26,31]. As such it provides an incidence function of the rate of organism movement through every pixel in the landscape as a function of the density and number of source points, the dispersal ability of the species, and the resistance of the landscape [55]. It also generates a spatial incidence function for each species’ predicted rate of movement across each pixel in the landscape [66].

To account for uncertainties regarding movement behavior and reliable dispersal data for both species in Iran and evaluate how robust our predicted core habitats are to this uncertainty, we ran a sensitivity analysis. Hence, we analyzed Four distance thresholds (the maximum distance any species in the region can be moved or disseminated) including: 50000, 100000, 150000 and 200000 cost units, which represent movement abilities of 50, 100, 150 and 200 km, respectively, through optimum, low resistance habitat [9]. This is important given research that suggests that dispersal ability is among the most influential factors on predicted functional connectivity, and often much more influential than landscape resistance itself [67].

We used the resistant kernel connectivity maps to identify core areas for each species [68,69]. We defined core habitat patches as contiguous patches with resistant kernel values > 10% of the highest recorded for the species [66,70]. We ranked these key patches based on their strength (sum of kernel values) and size [12]. The final ranking value for the core areas prioritization represented the averaged values of these sub-rankings. We quantified the extent and percentage of CAs and corridors for each species that were within the current conservation network to evaluate the effectiveness of the current conservation network in providing connectivity for these species in the study area. We also intersected both study species’ projected core habitats and corridor paths to identify important areas for both species [71].

To evaluate the differences in the spatial pattern and configuration of habitat, we calculated a suite of fragmentation metrics with FRAGSTATS [72]. For each species, all values above the 10th percentile of the highest dispersal scenario were reclassified as 1, representing habitat patches of high connectivity. Everything else was reclassified as 0 [2]. Then, we calculated four class level metrics using FRAGSTATS v4.2.1 [73] including: (I) the percentage of the landscape (PLAND), which quantifies the habitat patches of high connectivity as a percentage of the study area; (II) radius of gyration (GYRATE_AM) or correlation length, which provides a measurement of the extensiveness of habitat patches of high connectivity; (III) largest patch index (LPI), which represents the percentage of the landscape comprised by the largest habitat patch of high connectivity; (IV) number of isolated patches (NP). These metrics have been used frequently in past connectivity research [7,17,74,75].

### 2.5 Grey wolf and golden jackal vehicle collisions

Vehicle collision locations for grey wolf and golden jackal were obtained during 2013–2018 by first author through a field survey in collaboration with DoE rangers. Differences in the number of road-kill carcasses observed seasonally were compared by using a Kruskal-Wallis test [76] with seasonal data pooled across years. This nonparametric procedure was used to test for seasonal differences in the number of road-kills for both species.

### 2.6 Evaluating congruence between crossing points and predicted connectivity

We used a spatial randomization testing procedure to evaluate congruence between the locations where grey wolf and golden jackal were observed crossing the road [28]. Spatial randomization testing of this kind is recommended in cases where there is spatial dependence among observations and produces an unbiased estimate of the probability of the observed outcome given the data [28].

We compared the median value of predicted connectivity (resistant kernel surfaces at each dispersal ability) for the 170 golden jackal and 101 grey wolf crossing locations with the distribution of median values of 1 × 10^7^ random samples of 170 and 101 sites along the highway within the study area. Crossing locations of both species, were gathered by DoE rangers and first author of this research with random patrol monitoring during 2013–2018. For each combination of species and dispersal ability, we calculated the ranking of the median of observed values within the distribution of the medians of the 1×10^7^ random samples.). Crossing locations are areas that species were observed to cross the road.

## 3. Results

### 3.1 Predicted distribution of grey wolf and golden jackal

Among all the model’s RF and GLM represented the highest and lowest performance in predicting habitat suitability for both species, respectively (Table 1). For grey wolf, elevation, topographic ruggedness and distance to CAs were the strongest variables for predicting the target species’ distribution. For golden jackal, distance to human settlements, dump sites and topographic roughness were the most important variables predicting occurrence in the study area (S3 Table).

**Table 1:** Accuracy evaluation of the different models’ models (TSS, AUC) used to predict distribution of grey wolf and golden jackal in central Iran. Republished from [http://www.frw.ir] under a CC BY license, with permission from [Forest, Range, Watershed Management Organization of Markazi province (IFRWO)], original copyright [2021].

Model	Grey wolf		Golden jackal	
	TSS	AUC	TSS	AUC
GLM	0.530	0.650	0.530	0.690
GBM	0.610	0.810	0.640	0.790
MAXENT	0.780	0.866	0.790	0.840
RF	0.950	0.980	0.910	0.920
MARS	0.590	0.680	0.599	0.710

Golden jackal showed relatively complex non-linear relationships with most predictor variables (S2 Fig). There was a clear negative association with increasing distance to dumpsites and increasing topographical roughness (at the lower end of these variables), suggesting that jackals are often found in association with proximity to dump sites in flat terrain. However, there seems to be a multimodal habitat niche in relation to these variables, with occurrence also increasing somewhat in areas far from dump sites with relatively high topographical ruggedness This species had no clear association with distance to CAs. This suggests that jackal is likely a species with a plastic habitat niche that can shift in different landscape contexts to utilize different resources.

In contrast to jackal, grey wolf clear and strong associations with distance to human settlements, distance to CAs, elevation and NDVI, suggesting this species is most commonly found in more productive ecosystems at higher elevations and farther from human settlements (S3 Fig). There seems, however, to be a negative association with ruggedness, suggesting, that while wolf select areas of higher elevation far from human settlements, they select areas in those regions that are topographically relatively flat. Also, in contrast to jackal, wolf had no clear association with distance to dump sites.

**Fig 2:**
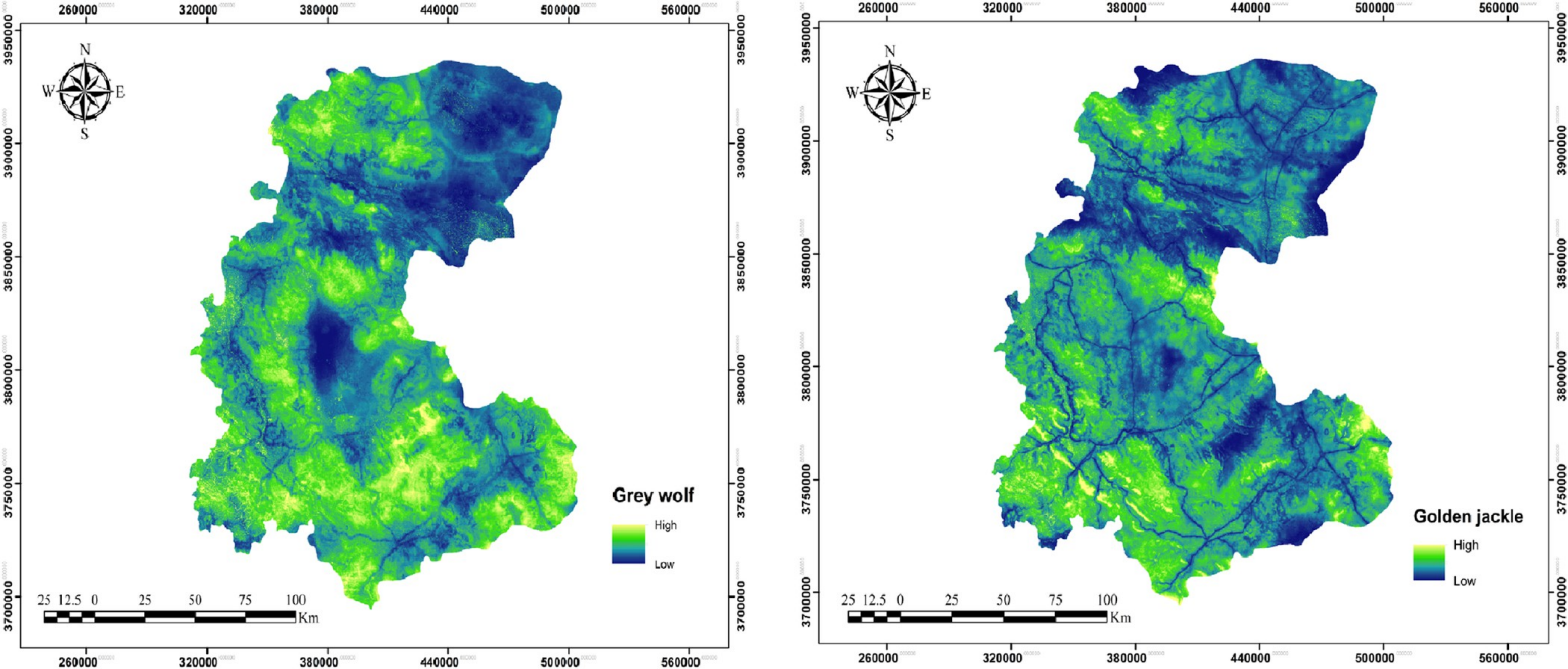
Predicted suitability of the study area for grey wolf and golden jackal based on the combined result of five SDMs. Republished from [http://www.frw.ir] under a CC BY license, with permission from [Forest, Range, Watershed Management Organization of Markazi province (IFRWO)], original copyright [2021].

The ensemble models’ predictions for grey wolf and golden jackal showed that large areas of the landscape could support the occurrence of both species (Fig 2). However, the predicted suitable areas for gray wolf were more concentrated and spatially demarcated. 75 percent of the area was predicted to be suitable for grey wolf and slightly less (74%) for golden jackal (S4 Fig, 1 means high suitable habitat and 0 means less suitable habitat). 65% of suitable habitats of both species were spatially overlapping.

### 3.2 Core habitat and connectivity

**Fig 3:**
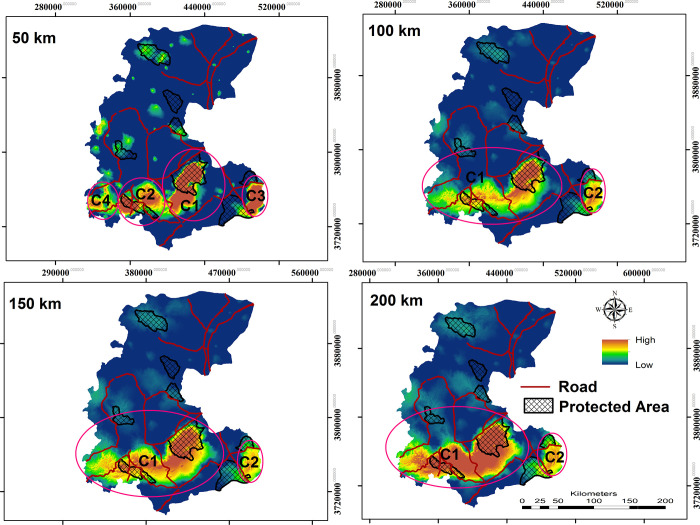
Grey wolf core areas at dispersal ability 50, 100, 150 and 200 km respectively and network of CAs and roads. Republished from [http://www.frw.ir] under a CC BY license, with permission from [Forest, Range, Watershed Management Organization of Markazi province (IFRWO)], original copyright [2021]. Republished from [https://markazi.doe.ir/] under a CC BY license, with permission from [Markazi Province Office of Department of Environment (DOE)], original copyright [2021].

**Fig 4:**
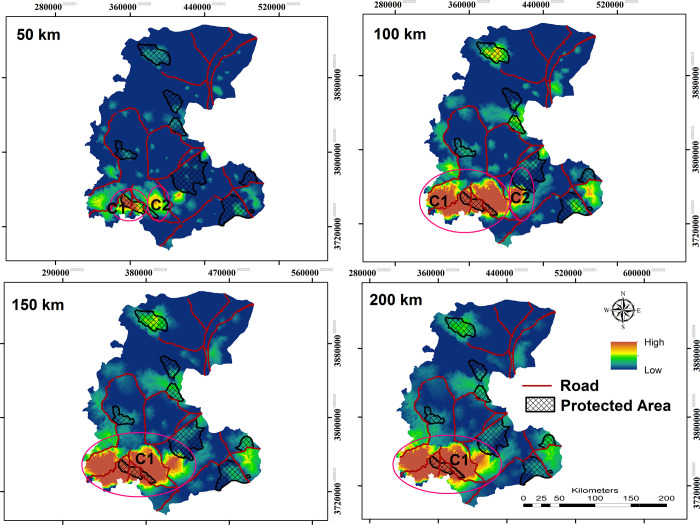
Golden Jackal core areas at dispersal ability 50, 100, 150 and 200 km respectively and network of CAs and roads. Republished from [http://www.frw.ir] under a CC BY license, with permission from [Forest, Range, Watershed Management Organization of Markazi province (IFRWO)], original copyright [2021]. Republished from [https://markazi.doe.ir/] under a CC BY license, with permission from [Markazi Province Office of Department of Environment (DOE)], original copyright [2021].

Our connectivity simulation modeling for grey wolf revealed that core habitats are large and concentrated in the study area’s southern regions. Among the identified core habitat patches, eight are larger than 2000 km^2^. The largest and most important core area (C1 in Fig 3) is of 49800 km^2^ and is located in the southern part of the landscape (Fig 3). The second largest and most important core area, based on size (6200 km^2^) and strength (sum of kernel value), occurred in the southwestern part of the landscape (Haftadgholleh and Alvand Protected Areas and Rasvand Wildlife Refuge). Also, an average of 37.84% of the identified core habitats for the grey wolf is covered by CAs. The highest overlap between core habitats and CAs was observed in the southern part of the landscape which three CAs (Haftadgholleh and Alvand Protected Areas and Rasvand Wildlife Refuge) were covered by the most important identified core areas. The largest and most important core area (C1 in Fig 4) for golden jackal, according to size (38800.20 km^2^) and strength (sum of kernel value), is in the southern part of the study area (Fig 4 and Table 2). For this species, 18.6% of the predicted core habitats are covered by CAs.

**Table 2:** The extent and percent of core habitats covered by current conservation networks for grey wolf and golden jackal in Central Iran. The median value of habitat suitability for presence points was used as the threshold to define the highly suitable habitats.

Dispersal abilities for each species	Extent of core habitats (km^2^)	Extent of protected core habitats (km^2^)	% of protected core habitats
Grey wolf			
50 km	5059.04	1493.13	40.51
100 km	6885.17	2003.87	39.10
150 km	7953.80	2224.07	38.96
200km	8976.32	2318.49	32.82
Golden jackal			
50 km	3974.24	871.22	21
100 km	5764.71	1120.83	19.44
150 km	7101.28	1313.13	18.49
200km	8545.13	1322.65	15.47

**Fig 5:**
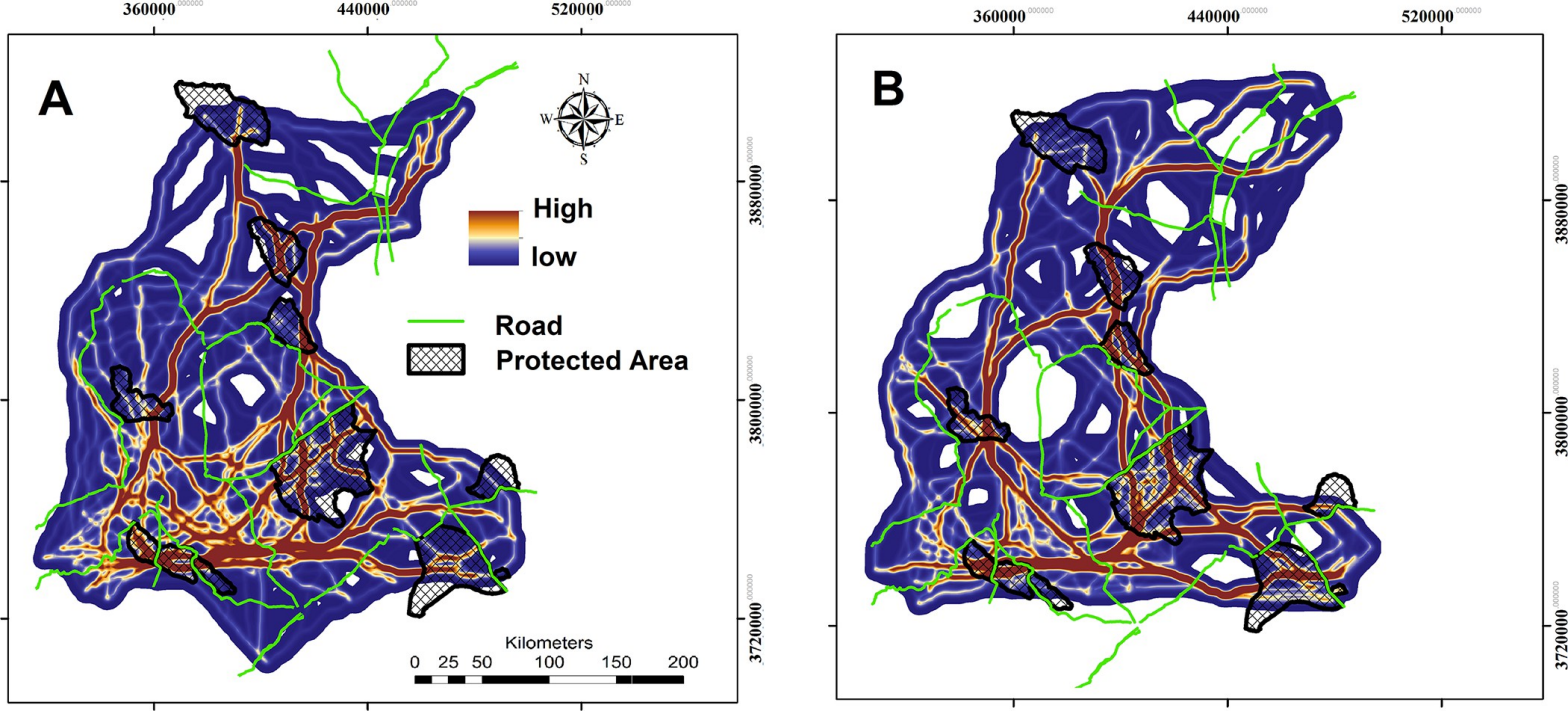
UNICOR corridor pathways for the golden jackal (A) and the grey wolf (B) in Central Iran. Republished from [http://www.frw.ir] under a CC BY license, with permission from [Forest, Range, Watershed Management Organization of Markazi province (IFRWO)], original copyright [2021]. Republished from [https://markazi.doe.ir/] under a CC BY license, with permission from [Markazi Province Office of Department of Environment (DOE)], original copyright [2021].

In the southern sections of the study region, our grey wolf connectivity simulation modeling showed a high level of habitat connectivity. A total of 25.18% of the extent of this corridor network is covered with CAs (Fig 5 and Table 3). Most of the identified corridor networks for the golden jackal also occurred in the southern parts of the study area. Of the predicted corridor paths of the species, 19.11% are covered by CAs (Fig 5 and Table 3). Our analysis showed that most predicted corridor paths for both species are bisected multiple times by roads (Fig 5 and Table 3).

**Table 3:** The extent and percent of corridors covered by current conservation networks for golden jackal and grey wolf in Central Iran. The median value of habitat suitability for presence points was used as threshold to define the highly suitable habitats.

Species	Extent of corridors (km^2^)	Extent of protected corridors (km^2^)	% of protected corridors	Length of paved road cross the corridor path (km)
Grey wolf	3725.76	938.42	25.18	149.91
Golden jackal	3387.85	647.51	19.11	119.22

There was one core habitat that was shared by the two study species (S5 Fig). 68.59% of the total extent of predicted core habitats overlapped between the two species. The southern and central sections of the study area had the highest predicted connectivity for both species (S5 Fig). Around 33 percent (32.67%) of corridors of both species were overlapping (S5 Fig).

### 3.3 Landscape connectivity four levels of dispersal abilities

For both species, the percentage of the landscape, correlation length and largest patch index of connected habitat was predicted to increase significantly, and the number of patches was predicted to decrease, with increasing dispersal ability (Table 4). Across the four dispersal thresholds and different models, we predict that between 17% and 30% of the landscape contains connected habitat patches for gray wolf. For golden jackal, 13% to 29% of the landscape contains connected habitat patches. We predicted that isolated patches for grey wolf and golden jackal ranged between 4–35 and 9–49 respectively across dispersal thresholds and modeling methods (Table 4).

**Table 4:** FRAGSTATS results for four metrics includes: Number of individual core patches (NP) largest patch index (LPI), percentage of landscape in connected habitat (PLAND) and correlation length of core habitats (CL). For grey wolf and golden jackal in four levels of dispersal ability (50,000, 100,000, 150,000 and 200000). The core habitats were defined as contiguous units with resistant kernel values >10% of the highest resistance kernel for the species.

Species	Dispersal ability	NP	LPI	PLAND	CL
Grey wolf	50	35	11.95	17.36	2823.44
	100	24	17.27	23.63	3165.37
	150	6	20.31	27.30	12316.13
	200	4	23.06	30.81	18091.81
Golden Jackal	50	49	8.39	13.64	2271.65
	100	40	13.16	19.79	2263.34
	150	10	15.88	24.37	8274.41
	200	9	19.82	29.33	9404.16

### 3.4 Spatial randomization test

During the study period (2013–2018), 173 golden jackal and 103 wolf vehicle collisions were recorded. Most golden jackal road mortalities occurred in the spring (36.41%) and winter seasons (26.58%), while for grey wolf most collisions occurred in summer (19.41%) and winter (33.98%) seasons (Table 5). Seasonal variation in road-kills was not evident for grey wolf (X^2^ (2) = 3.06, P 0.480) and golden jackal (X^2^ (2) = 3.7, P = 0.256), We also gathered 101 and 170 additional gray wolf and golden jackal crossing locations, respectively from observation.

**Table 5:** Annual distribution of road mortality for the two study species *Canis lupus* and *C*. *aureus*, on the Markazi Province’s main and secondary roads (Iran).

Species	season	2013	2014	2015	2016	2017	2018	Total
Golden jackal	Spring	20	13	5	10	7	8	63
	Summer	10	10	4	3	7	7	41
	Autumn	8	9	1	2	0	3	23
	Winter	13	15	3	9	1	5	46
	**Total**	51	44	13	24	15	23	
Grey wolf	Spring	4	4	5	1	3	5	24
	Summer	4	3	4	10	4	2	27
	Autumn	5	2	2	3	5	2	19
	Winter	9	6	8	5	5	2	35
	**Total**	20	15	19	19	17	11	

**Fig 6:**
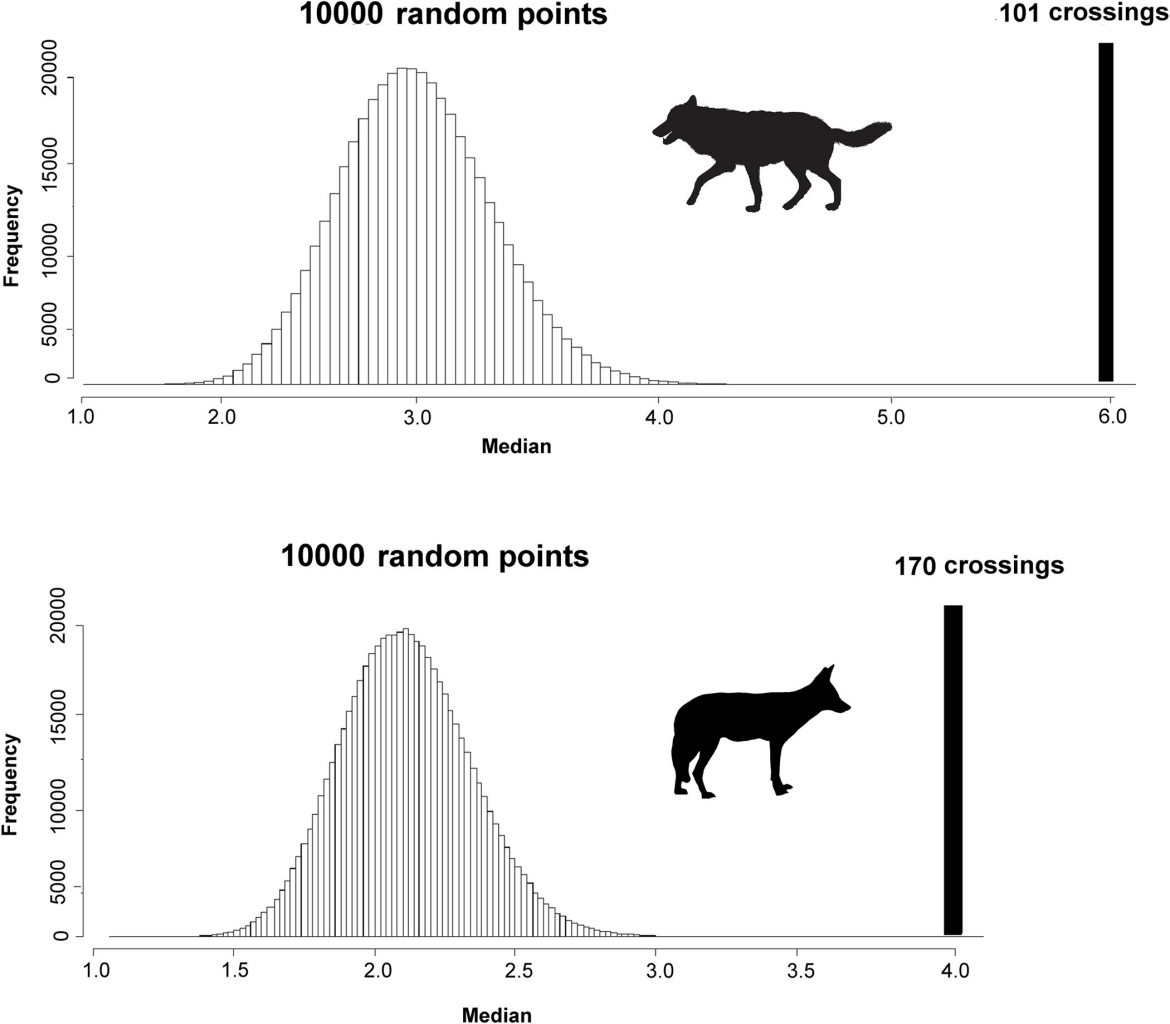
Spatial randomization test: The crossing location of grey wolf (above) and golden jackal (below) has a much higher connectivity score than the randomization. A solid vertical line shows the median of 101and 170 crossing locations for grey wolf and golden jackal respectively. Transparent bars show the distribution of the median connectivity values of 10000 random spatial samples across the road network.

We found our connectivity model very strongly predicted grey wolf and golden jackal (Fig 6) highway crossing locations (Table 6). Crossings have a significantly higher connectivity score than the randomly-selected locations (P < 0.00001).

**Table 6:** Maximum, minimum, median, and average value of grey wolf and golden jackal crossing locations compare with 10000 random points.

	Crossing locations				
species	Dispersal ability	Max	Min	Median	Average
Grey wolf	50	22	1.5	5	5.50
	100	24	3.2	6	6.80
	150	32	3.6	6	7.30
	200	32	3.6	6	8.80
Golden jackal	50	20	1.2	3	4.80
	100	23	2.2	4	5.55
	150	30	3.1	4	7.80
	200	30	3.2	4	8.10
Random points					
species	Dispersal ability	Max	Min	Median	Average
Grey wolf	50	10	0	0.65	2.12
	100	15	0	0.94	2.13
	150	15	0.25	1.2	2.5
	200	19	0.60	0.80	3.5
Golden jackal	50	11	0	0.36	1.6
	100	12	0	0.75	2.06
	150	14	0.55	0.89	2.50
	200	17	0.80	1.30	2.90

## 4. Discussion

Grey wolf and golden jackal are sympatric in Iran, making them good candidate species for comparative studies of habitat selection, distribution and connectivity. The habitat requirements of grey wolf and golden jackal also deserve attention because they generally require large home ranges, are negatively impacted by changes in land use and are killed because of the threats they pose to livelihoods [48,77].

In this study, we provided the first assessment of habitat suitability and ecological connectivity for the golden jackal and grey wolf in a human dominated landscape in central Iran. Our results identify areas of the landscape that would be most effective for multiple species conservation of habitat quality and connectivity for both species.

### 4.1 Influence of environmental variables on grey wolf and golden jackal potential distribution

Our results identify important niche differentiation of grey wolf and golden jackal in central Iran. Specifically, golden jackal is seen to have complex, nonlinear relationships with several predictor variables, indicating ecological plasticity which enables it to utilize different resources and conditions in different parts of the landscape. Specifically, golden jackal is associated with areas close to dump sites, with low topographical ruggedness and low vegetation productivity. In these areas, jackals are supported in a scavenger role associated with food resources provided by human dump sites, which are generally in lower elevation areas with relatively low vegetation productivity. This is consistent with our hypotheses. However, our models also found positive association of jackals with areas of higher elevation, higher topographical ruggedness and higher values of NDVI (vegetation productivity), suggesting that they also are associated with more productive ecosystems with lower human disturbance. Golden jackal is highly adapted to live in human-dominated landscapes where they take advantage of various anthropogenic resources [78–80], however, our findings revealed that they generally are found with higher probability in areas more distant from human occupation, with the exception of dump sites. Consistent with our findings, Yusefi et al. (2021) showed that this species found in an area with low human population [41]. Our findings revealed that growing distance to dumpsites reduces the occurrence of this species, which is consistent with this species’ scavenging behavior. Most road mortalities were recorded in the southern parts of the study area. This could be due to the greater traffic volume on the southern highways and to the high concentration of villages and rural areas in the southern parts resulting in increased food resource availability such as dumpsites [81,82].

In contrast to golden jackal, the distribution of the grey wolf was predominantly influenced by anthropogenic and topographical factors (e.g., distance to settlements, roads, elevation and ruggedness), a finding reported for other carnivore species in Iran [2,51,83]. Grey wolf had clear positive associations with distance to human settlements, increasing elevation and increasing vegetation productivity. This suggests that wolves avoid areas of high risk of anthropogenic disturbance, selecting areas farther from settlements, at higher elevations, which also have higher precipitation and density of prey species. This is consistent with our hypotheses. In contrast to our finding Mohammadi et al (2021) showed that grey wolf had higher occupancy near villages. Moreover, their result showed that grey wolf had no relationship with elevation [84]. In contrast to jackal, wolves had not clear association with distance to dump sites, and a moderately increasing rate of occurrence with increasing distance to roads. Ahmadi et al. (2014) have suggested that grey wolves can persist in mixed agroecosystems due to the spatial heterogeneity in human activities [51]. Our findings, on the other hand, indicate a negative relationship of wolves with settlements and roads. The results of our model suggested that with increasing distance to road, wolf presence increased [48,85–87]. This finding indicates that wolves may displace activity to avoid coming into contact with humans by choosing higher elevation and rougher terrain away from roads [48]. Our results in this regard are similar to Ahmadi et al. (2014) [51] who showed that areas selected by wolves for denning were characterized by low density of settlements and primary roads. Their result showed that wolves primarily establish dens in the sides of elevated steep-slope hills. Our results also revealed that elevation and ruggedness were important predictors of wolf presence. Also, our results showed that habitat suitability of both species is highly overlapping in areas of high vegetation productivity away from settlements, which is in accordance with our first hypothesis.

### 4.2 Connectivity networks and core habitat distribution

Effective management of large carnivores requires identifying core habitats and corridor networks between them [2,9,12,88]. According to our connectivity analysis the southern parts of the study area were predicted to contain the largest extent of potentially suitable habitats for both target species (Fig 5). In the southern part of the landscape, we found four and two critical core habitats for grey wolves and golden jackals, respectively (Figs 3 to 5). High connectivity areas in the southern parts of the study area are predicted to connect these core habitats (S5 Fig). Our result showed that C1 at larger dispersal abilities was the most important core area for both species. Considering this core area as the most important area of the landscape for conservation to foster network connectivity is critical in this regard.

Our resistant kernel analysis showed that between 32–41% of identified core habitats for grey wolf are covered by CAs, depending on dispersal ability. For the golden jackal we found a lower contribution of the CAs as core habitats (15–21%), which was explained by species’ stronger association with human dominated landscapes. For this canid, the highest overlap between core habitats and conservation areas was observed for C1 and C2, incorporating a considerable number of villages. In contrast, the highest overlap between grey wolf core habitats and Conservation Areas was observed for core numbers C1, C2, C3 and C4 (Fig 4), with 40% of core habitats intersecting with the CAs (Fig 4).

Most CAs networks in Iran, are fragmented by roads, and road collisions present a serious threat for carnivores [89]. We identified vulnerable parts of the connectivity network in the southern part of the study area (C1 and C2) where roads intersected important movement corridors [28]. The vulnerability of these locations is related to the high potential for grey wolf and golden jackal vehicle collisions. Our findings are similar to Moqanaki and Cushman (2017) [29] and Mohammadi et al (2021) [90] who showed that core areas for Asiatic cheetah and Persian onager and the corridors that link them are bisected by multiple primary and secondary roads.

One of our study’s most novel aspects is the validation of our predicted connectivity maps with independent data on road mortality and crossing locations of both species. Relatively few studies have independently validated connectivity predictions with movement [28], density [91] mortality, or genetic data [63,92]. The predicted connectivity value of our connectivity models was strongly associated with the locations of observed road crossing for both species, as shown by our spatial randomization method. Predicted connectivity is highly related to the actual patterns of observed road mortality and crossing in the study area for both species, providing independent validation of our predictions. This significantly strengthens their utility for decision-making. The lack of use of GPS telemetry to capture coherent and accurate occurrence locations of both species is one of the study’s limitation. Furthermore, inaccuracies in predicting landscape resistance can have a significant impact on population connectivity forecasts [28]. As a result, it is critical that the findings we have uncovered here be confirmed, validated, and modified using more reliable approaches for assessing connection, such as landscape genetics [75,93].

## 5. Conclusion

The research presented here focused on identifying suitable habitat, the most important core areas, the strongest potential corridors that connect them and validating these models with independent movement data for grey wolf and golden jackal. Based on our results we see that grey wolf in central Iran is more associated with areas of low human influence than previously believed, but that jackal has a broader, multimodal niche which includes areas close to human habitations (dump sites in particular). Our connectivity analyses identified the most important core areas and corridors for both species. These overlapped to a considerable degree in higher elevation areas of high vegetation productivity away from human settlements and roads. Based on our results we recommend: (1) conserving the core area patches we identified in unprotected lands in important core areas (particularly C1 and C2), (2) protecting and enhancing habitat quality and connectivity along important corridors between core area patches, particularly between C1 and C2; and (3) implementing mitigating measures for reducing grey wolf and golden jackal vehicle collisions, especially in main movement corridors in the southern part of the study area.

## Supporting information

S1 Fig Global Moran’s I test. (DOCX)

S2 Fig Random forest partial dependence plot response curves of presence points to the environmental variables in habitat modeling of the golden jackal in central Iran. We only showed the output of this model because among all the models, RF represented the highest performance in predicting habitat suitability for this species. Republished from [http://www.frw.ir] under a CC BY license, with permission from [Forest, Range, Watershed Management Organization of Markazi province (IFRWO)], original copyright [2021]. Republished from [https://markazi.doe.ir/] under a CC BY license, with permission from [Markazi Province Office of Department of Environment (DOE)], original copyright [2021].
(DOCX)

S3 Fig Random forest partial dependence plot response curves of presence points to the environmental variables in habitat modeling of the grey wolf in central Iran. We only provided the output of this model because among all the models, RF represented the highest performance in predicting habitat suitability for this species. Republished from [http://www.frw.ir] under a CC BY license, with permission from [Forest, Range, Watershed Management Organization of Markazi province (IFRWO)], original copyright [2021]. Republished from [https://markazi.doe.ir/] under a CC BY license, with permission from [Markazi Province Office of Department of Environment (DOE)], original copyright [2021].
(DOCX)

S4 Fig Predicted binary suitability of the study area for a: Grey wolf and b: Golden jackal based on the combined result of five SDMs. Contains information from OpenStreetMap and OpenStreetMap Foundation, which is made available under the Open Database License. Republished from [http://www.frw.ir] under a CC BY license, with permission from [Forest, Range, Watershed Management Organization of Markazi province (IFRWO)], original copyright [2021].
(DOCX)

S5 Fig Intersection map for predicted core habitats (A) and corridors (B) of grey wolf and golden jackal in Central of Iran. The colors depict different species connectivity. Contains information from OpenStreetMap and OpenStreetMap Foundation, which is made available under the Open Database License. Republished from [https://markazi.doe.ir/] under a CC BY license, with permission from [Markazi Province Office of Department of Environment (DOE)], original copyright [2021].
(DOCX)

S1 Table Presence points of golden jackal (*Canis aureus*) and grey wolf (*Canis lupus*) in Markazi province (2000–2019). (DOCX)

S2 Table Environmental and anthropogenic variables and their sources. (DOCX)

S3 Table Variable contribution in the habitat modeling of the grey wolf and golden jackal in central Iran. Republished from [http://www.frw.ir] under a CC BY license, with permission from [Forest, Range, Watershed Management Organization of Markazi province (IFRWO)], original copyright [2021]. Republished from [https://markazi.doe.ir/] under a CC BY license, with permission from [Markazi Province Office of Department of Environment (DOE)], original copyright [2021].
(DOCX)
