# Correction: Identifying connectivity for two sympatric carnivores in human-dominated landscapes in central Iran

**DOI:** 10.1371/journal.pone.0293794

**Published:** 2023-10-26

**Authors:** Sahar Rezaei, Alireza Mohammadi, Roberta Bencini, Thomas Rooney, Morteza Naderi

The affiliation for the last author is incorrect. The correct affiliation for Morteza Naderi is Arak University.

An additional affiliation is missing for the last author. Morteza Naderi is also affiliated with the Department of Molecular Biology and Genetics, Faculty of Sciences, Koc University, Istanbul, Turkey.

## References

[1] Rezaei S, Mohammadi A, Bencini R, Rooney T, Naderi M. (2022) Identifying connectivity for two sympatric carnivores in human-dominated landscapes in central Iran. PloS ONE 17(6): e0269179. 10.1371/journal.pone.0269179 35709185 PMC9202930

