# Supplementary figures and images for: Identifying connectivity for two sympatric carnivores in human-dominated landscapes in central Iran
Source: PLoS One. 2022 Jun 16;17(6):e0269179. doi: 10.1371/journal.pone.0269179 (PMC9202930; doi:10.1371/journal.pone.0269179)

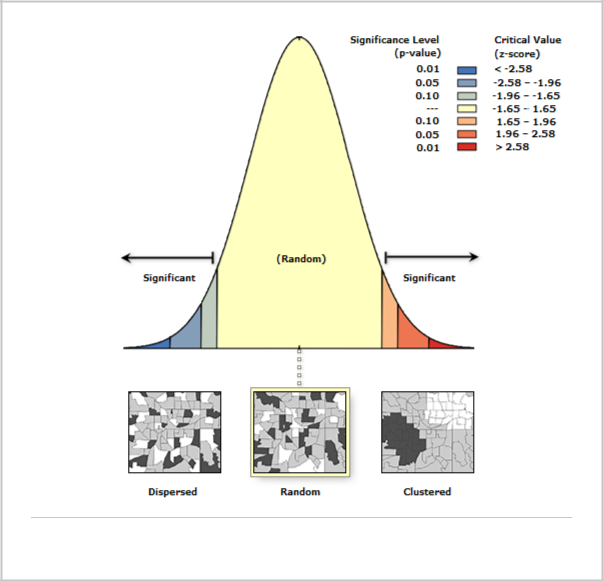


Figure S1: global Moran’s I test.

Supplement: S1 Fig — (DOCX) [file pone.0269179.s001.docx]
