# Supplementary material for: Identifying connectivity for two sympatric carnivores in human-dominated landscapes in central Iran
Source: PLoS One. 2022 Jun 16;17(6):e0269179. doi: 10.1371/journal.pone.0269179 (PMC9202930; doi:10.1371/journal.pone.0269179)
Supplement: S3 Fig — We only provided the output of this model because among all the models, RF represented the highest performance in predicting habitat suitability for this species. Republished from [http://www.frw.ir] under a CC BY license, with permission from [Forest, Range, Watershed Management Organization of Markazi province (IFRWO)], original copyright [2021]. Republished from [https://markazi.doe.ir/] under a CC BY license, with permission from [Markazi Province Office of Department of Environment (DOE)], original copyright [2021]. (DOCX) [file pone.0269179.s003.docx]

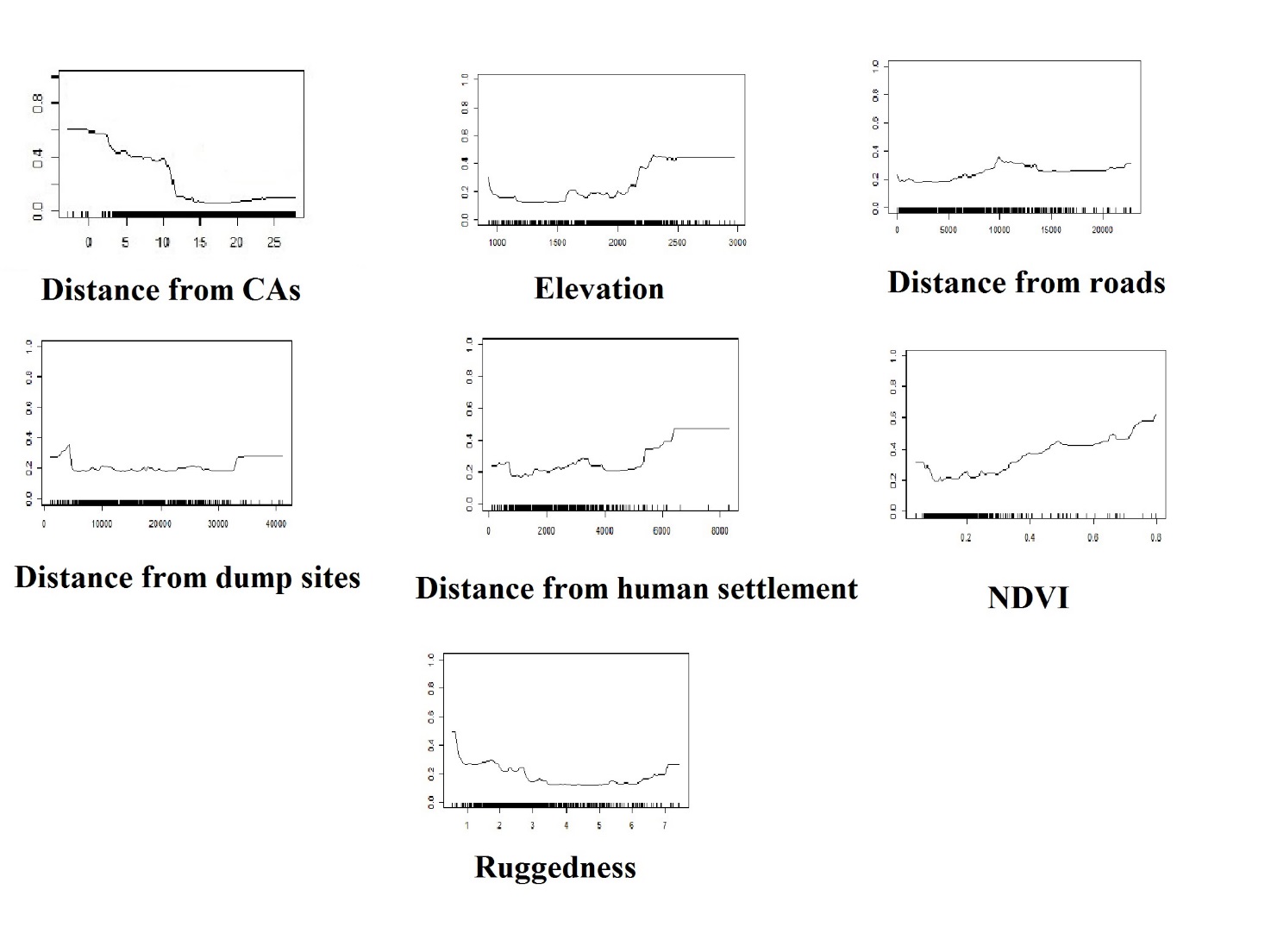


Figure S3. Random forest partial dependence plot response curves of presence points to the environmental variables in habitat modeling of the grey wolf in central Iran. We only provided the output of this model because among all the models, RF represented the highest performance in predicting habitat suitability for this species. Republished from [ [http://www.frw.ir](http://www.frw.ir/)] under a CC BY license, with permission from [Forest, Range, Watershed Management Organization of Markazi province (IFRWO)], original copyright [2021]. Republished from [ https://markazi.doe.ir/] under a CC BY license, with permission from [Markazi Province Office of Department of Environment (DOE)], original copyright [2021].
