# Supplementary material for: Identifying connectivity for two sympatric carnivores in human-dominated landscapes in central Iran
Source: PLoS One. 2022 Jun 16;17(6):e0269179. doi: 10.1371/journal.pone.0269179 (PMC9202930; doi:10.1371/journal.pone.0269179)
Supplement: S4 Fig — Contains information from OpenStreetMap and OpenStreetMap Foundation, which is made available under the Open Database License. Republished from [http://www.frw.ir] under a CC BY license, with permission from [Forest, Range, Watershed Management Organization of Markazi province (IFRWO)], original copyright [2021]. (DOCX) [file pone.0269179.s004.docx]

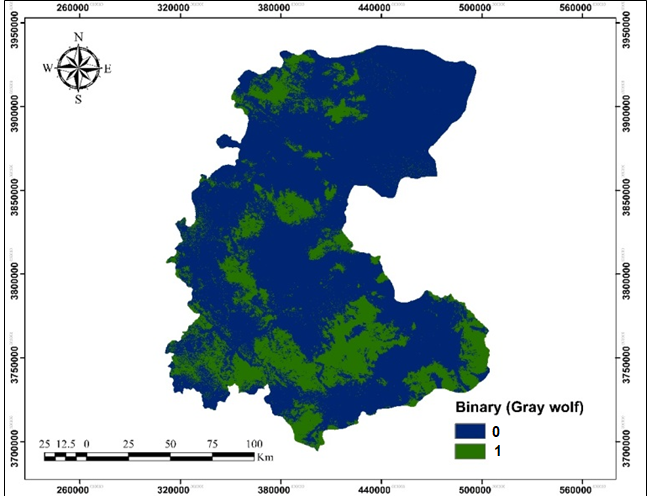


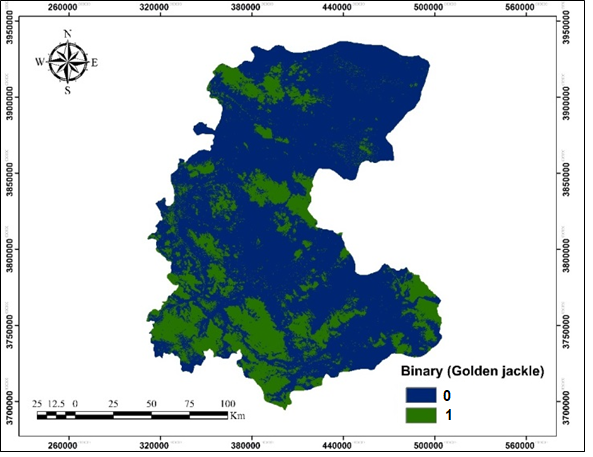


Figure S4. Predicted binary suitability of the study area for a: grey wolf and b: golden jackal based on the combined result of five SDMs. Contains information from OpenStreetMap and OpenStreetMap Foundation, which is made available under the Open Database License. Republished from [ [http://www.frw.ir](http://www.frw.ir/)] under a CC BY license, with permission from [Forest, Range, Watershed Management Organization of Markazi province (IFRWO)], original copyright [2021].
