# Supplementary material for: Identifying connectivity for two sympatric carnivores in human-dominated landscapes in central Iran
Source: PLoS One. 2022 Jun 16;17(6):e0269179. doi: 10.1371/journal.pone.0269179 (PMC9202930; doi:10.1371/journal.pone.0269179)
Supplement: S5 Fig — Intersection map for predicted core habitats (A) and corridors (B) of grey wolf and golden jackal in Central of Iran. The colors depict different species connectivity. Contains information from OpenStreetMap and OpenStreetMap Foundation, which is made available under the Open Database License. Republished from [https://markazi.doe.ir/] under a CC BY license, with permission from [Markazi Province Office of Department of Environment (DOE)], original copyright [2021]. (DOCX) [file pone.0269179.s005.docx]

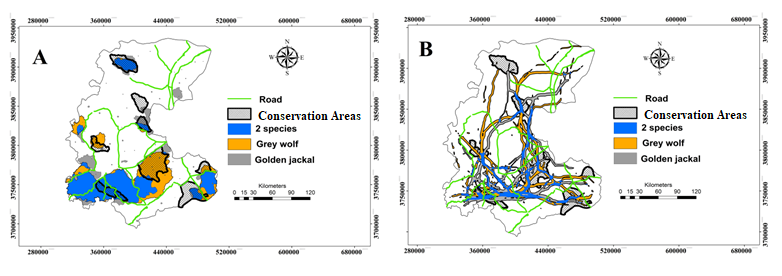


Figure S5. Intersection map for predicted core habitats (A) and corridors (B) of grey wolf and golden jackal in Central of Iran. The colors depict different species connectivity. Contains information from OpenStreetMap and OpenStreetMap Foundation, which is made available under the Open Database License. Republished from [ https://markazi.doe.ir/] under a CC BY license, with permission from [Markazi Province Office of Department of Environment (DOE)], original copyright [2021].
