# Supplementary material for: Identifying connectivity for two sympatric carnivores in human-dominated landscapes in central Iran
Source: PLoS One. 2022 Jun 16;17(6):e0269179. doi: 10.1371/journal.pone.0269179 (PMC9202930; doi:10.1371/journal.pone.0269179)
Supplement: S1 Table — (DOCX) [file pone.0269179.s006.docx]

Table S1: Presence points of golden jackal (*Canis aureus*) and grey wolf (*Canis lupus*) in Markazi province (2000-2019).

| \| species \| x \| \| y \| \| --- \| --- \| --- \| --- \| \| jackal \| 442134 \| 3793640 \| \| \| jackal \| 440061 \| 3794210 \| \| \| jackal \| 440115 \| 3798690 \| \| \| jackal \| 362092 \| 3741260 \| \| \| jackal \| 404379 \| 3830690 \| \| \| jackal \| 378915 \| 3904200 \| \| \| jackal \| 389973 \| 3779860 \| \| \| jackal \| 357486 \| 3795800 \| \| \| jackal \| 414571 \| 3757460 \| \| \| jackal \| 414827 \| 3754320 \| \| \| jackal \| 356297 \| 3744680 \| \| \| jackal \| 353981 \| 3748730 \| \| \| jackal \| 355473 \| 3747900 \| \| \| jackal \| 450961 \| 3733540 \| \| \| jackal \| 480836 \| 3734970 \| \| \| jackal \| 495046 \| 3742060 \| \| \| jackal \| 495462 \| 3749020 \| \| \| jackal \| 468885 \| 3757060 \| \| \| jackal \| 490810 \| 3755870 \| \| \| jackal \| 455595 \| 3749010 \| \| \| jackal \| 483116 \| 3758850 \| \| \| jackal \| 474658 \| 3738280 \| \| \| jackal \| 474710 \| 3772600 \| \| \| jackal \| 446802 \| 3770260 \| \| \| jackal \| 438028 \| 3762250 \| \| \| jackal \| 448417 \| 3779260 \| \| \| jackal \| 421983 \| 3772670 \| \| \| jackal \| 431465 \| 3784300 \| \| \| jackal \| 425753 \| 3742790 \| \| \| jackal \| 417297 \| 3749340 \| \| \| jackal \| 411045 \| 3735300 \| \| \| jackal \| 404891 \| 3738160 \| \| \| jackal \| 403853 \| 3744840 \| \| \| jackal \| 397745 \| 3736010 \| \| \| jackal \| 389994 \| 3740370 \| \| \| jackal \| 387440 \| 3745150 \| \| \| jackal \| 394093 \| 3749950 \| \| \| jackal \| 380833 \| 3736660 \| \| \| jackal \| 377360 \| 3751310 \| \| \| jackal \| 385680 \| 3753290 \| \| \| jackal \| 369454 \| 3758890 \| \| \| jackal \| 381826 \| 3764500 \| \| \| jackal \| 401189 \| 3754330 \| \| \| jackal \| 353283 \| 3737050 \| \| \| jackal \| 338175 \| 3731520 \| \| \| jackal \| 350059 \| 3746550 \| \| \| jackal \| 333697 \| 3736300 \| \| \| jackal \| 330109 \| 3739720 \| \| \| jackal \| 342213 \| 3727040 \| \| \| jackal \| 334293 \| 3729450 \| \| \| jackal \| 318708 \| 3741770 \| \| \| jackal \| 325397 \| 3745060 \| \| \| jackal \| 332365 \| 3748760 \| \| \| jackal \| 333329 \| 3758500 \| \| \| jackal \| 357368 \| 3757470 \| \| \| jackal \| 351280 \| 3761700 \| \| \| jackal \| 341778 \| 3773770 \| \| \| jackal \| 340891 \| 3780400 \| \| \| jackal \| 347350 \| 3782170 \| \| \| jackal \| 383289 \| 3780120 \| \| \| jackal \| 370175 \| 3775660 \| \| \| jackal \| 405415 \| 3777490 \| \| \| jackal \| 425873 \| 3801120 \| \| \| jackal \| 417549 \| 3813620 \| \| \| jackal \| 367871 \| 3802100 \| \| \| jackal \| 335996 \| 3805230 \| \| \| jackal \| 330854 \| 3814310 \| \| \| jackal \| 383179 \| 3823210 \| \| \| jackal \| 332110 \| 3824520 \| \| \| jackal \| 463050 \| 3903090 \| \| \| jackal \| 391251 \| 3891280 \| \| \| jackal \| 392879 \| 3907410 \| \| \| jackal \| 377825 \| 3836690 \| \| \| jackal \| 388505 \| 3896090 \| \| \| jackal \| 410530 \| 3845600 \| \| \| jackal \| 419227 \| 3785760 \| \| \| jackal \| 422911 \| 3768900 \| \| \| jackal \| 346712 \| 3759550 \| \| \| jackal \| 433494 \| 3726450 \| \| \| jackal \| 399400 \| 3793650 \| \| \| jackal \| 354709 \| 3841430 \| \| \| jackal \| 340760 \| 3849910 \| \| \| jackal \| 484264 \| 3747210 \| \| \| jackal \| 384382 \| 3806520 \| \| \| jackal \| 456299 \| 3863380 \| \| \| jackal \| 459617 \| 3896830 \| \| \| jackal \| 399878 \| 3897130 \| \| \| jackal \| 476520 \| 3906620 \| \| \| jackal \| 431435 \| 3859600 \| \| \| jackal \| 374209 \| 3734740 \| \| \| jackal \| 367111 \| 3738660 \| \| \| jackal \| 366920 \| 3742210 \| \| \| jackal \| 363395 \| 3743150 \| \| \| jackal \| 355321 \| 3741810 \| \| \| jackal \| 407772 \| 3747790 \| \| \| jackal \| 406479 \| 3754640 \| \| \| jackal \| 413246 \| 3752750 \| \| \| jackal \| 402408 \| 3703750 \| \| \| jackal \| 421274 \| 3868300 \| \| \| jackal \| 464571 \| 3885500 \| \| \| jackal \| 456681 \| 3879860 \| \| \| jackal \| 450578 \| 3867190 \| \| |
| --- | --- | --- | --- | --- | --- | --- | --- | --- | --- | --- | --- | --- | --- | --- | --- | --- | --- | --- | --- | --- | --- | --- | --- | --- | --- | --- | --- | --- | --- | --- | --- | --- | --- | --- | --- | --- | --- | --- | --- | --- | --- | --- | --- | --- | --- | --- | --- | --- | --- | --- | --- | --- | --- | --- | --- | --- | --- | --- | --- | --- | --- | --- | --- | --- | --- | --- | --- | --- | --- | --- | --- | --- | --- | --- | --- | --- | --- | --- | --- | --- | --- | --- | --- | --- | --- | --- | --- | --- | --- | --- | --- | --- | --- | --- | --- | --- | --- | --- | --- | --- | --- | --- | --- | --- | --- | --- | --- | --- | --- | --- | --- | --- | --- | --- | --- | --- | --- | --- | --- | --- | --- | --- | --- | --- | --- | --- | --- | --- | --- | --- | --- | --- | --- | --- | --- | --- | --- | --- | --- | --- | --- | --- | --- | --- | --- | --- | --- | --- | --- | --- | --- | --- | --- | --- | --- | --- | --- | --- | --- | --- | --- | --- | --- | --- | --- | --- | --- | --- | --- | --- | --- | --- | --- | --- | --- | --- | --- | --- | --- | --- | --- | --- | --- | --- | --- | --- | --- | --- | --- | --- | --- | --- | --- | --- | --- | --- | --- | --- | --- | --- | --- | --- | --- | --- | --- | --- | --- | --- | --- | --- | --- | --- | --- | --- | --- | --- | --- | --- | --- | --- | --- | --- | --- | --- | --- | --- | --- | --- | --- | --- | --- | --- | --- | --- | --- | --- | --- | --- | --- | --- | --- | --- | --- | --- | --- | --- | --- | --- | --- | --- | --- | --- | --- | --- | --- | --- | --- | --- | --- | --- | --- | --- | --- | --- | --- | --- | --- | --- | --- | --- | --- | --- | --- | --- | --- | --- | --- | --- | --- | --- | --- | --- | --- | --- | --- | --- | --- | --- | --- | --- | --- | --- | --- | --- | --- | --- | --- | --- | --- | --- | --- | --- | --- | --- | --- | --- | --- | --- | --- | --- | --- | --- | --- | --- | --- | --- | --- | --- | --- | --- | --- | --- | --- | --- | --- | --- | --- | --- | --- | --- | --- | --- | --- | --- | --- | --- | --- | --- | --- | --- | --- | --- | --- | --- | --- | --- | --- | --- | --- | --- | --- | --- | --- | --- | --- | --- | --- | --- | --- | --- | --- | --- | --- | --- | --- | --- | --- | --- | --- | --- | --- | --- | --- | --- | --- | --- | --- | --- | --- | --- | --- | --- | --- | --- | --- | --- | --- | --- | --- | --- | --- | --- | --- | --- | --- | --- | --- | --- | --- | --- | --- | --- | --- | --- | --- | --- | --- | --- | --- | --- | --- | --- |

| \| species \| x \| y \| \| --- \| --- \| --- \| \| wolf \| 368023.1 \| 3736578 \| \| wolf \| 359294.7 \| 3740823 \| \| wolf \| 360451.8 \| 3745029 \| \| wolf \| 357125 \| 3746403 \| \| wolf \| 354262.2 \| 3744239 \| \| wolf \| 354436.1 \| 3746007 \| \| wolf \| 392148.1 \| 3896528 \| \| wolf \| 396548.1 \| 3903247 \| \| wolf \| 377331.8 \| 3908325 \| \| wolf \| 435708.3 \| 3779494 \| \| wolf \| 429213.9 \| 3779574 \| \| wolf \| 430768.7 \| 3783616 \| \| wolf \| 435402.3 \| 3785976 \| \| wolf \| 386493.7 \| 3824305 \| \| wolf \| 427372.8 \| 3861905 \| \| wolf \| 363277.7 \| 3879433 \| \| wolf \| 384551.4 \| 3865353 \| \| wolf \| 389568.4 \| 3847929 \| \| wolf \| 358817.2 \| 3811077 \| \| wolf \| 326623.1 \| 3823695 \| \| wolf \| 333147.3 \| 3829781 \| \| wolf \| 345975.9 \| 3802457 \| \| wolf \| 335776.1 \| 3817766 \| \| wolf \| 327362.9 \| 3820332 \| \| wolf \| 325409.2 \| 3814974 \| \| wolf \| 323925.4 \| 3804435 \| \| wolf \| 337575.6 \| 3776430 \| \| wolf \| 323182.4 \| 3743610 \| \| wolf \| 317097.2 \| 3743700 \| \| wolf \| 333971.7 \| 3764200 \| \| wolf \| 350043.7 \| 3763054 \| \| wolf \| 358531.2 \| 3756568 \| \| wolf \| 332499 \| 3758640 \| \| wolf \| 334188.4 \| 3751299 \| \| wolf \| 331797.5 \| 3739136 \| \| wolf \| 337478.9 \| 3735831 \| \| wolf \| 333097.3 \| 3730763 \| \| wolf \| 345932.5 \| 3740730 \| \| wolf \| 384969.2 \| 3769427 \| \| wolf \| 371611.3 \| 3759832 \| \| wolf \| 378786.6 \| 3762611 \| \| wolf \| 378113.2 \| 3753729 \| \| wolf \| 392620.4 \| 3750223 \| \| wolf \| 399937.3 \| 3756464 \| \| wolf \| 385572 \| 3758019 \| \| wolf \| 376504.3 \| 3746355 \| \| wolf \| 386478 \| 3746844 \| \| wolf \| 382462.7 \| 3736793 \| \| wolf \| 396511.1 \| 3734203 \| \| wolf \| 406354.7 \| 3745111 \| \| wolf \| 404756.8 \| 3739835 \| \| wolf \| 411934.4 \| 3735602 \| \| wolf \| 414437.7 \| 3748224 \| \| wolf \| 428800 \| 3746208 \| \| wolf \| 419008.2 \| 3752069 \| \| wolf \| 421108.6 \| 3789376 \| \| wolf \| 438868.2 \| 3786830 \| \| wolf \| 427780.8 \| 3783423 \| \| wolf \| 421722.2 \| 3778936 \| \| wolf \| 421988 \| 3773676 \| \| wolf \| 414433 \| 3766000 \| \| wolf \| 427166.2 \| 3762841 \| \| wolf \| 488194.1 \| 3757950 \| \| wolf \| 496444.8 \| 3760010 \| \| wolf \| 491771.8 \| 3754100 \| \| wolf \| 502867 \| 3752366 \| \| wolf \| 497008.9 \| 3748993 \| \| wolf \| 492105.4 \| 3748302 \| \| wolf \| 481965.4 \| 3737036 \| \| wolf \| 492161.9 \| 3744495 \| \| wolf \| 487721.4 \| 3742026 \| \| wolf \| 492091.7 \| 3741410 \| \| wolf \| 493335.8 \| 3736153 \| \| wolf \| 488429.6 \| 3731953 \| \| wolf \| 481965.4 \| 3737036 \| \| wolf \| 470589.5 \| 3763954 \| \| wolf \| 457038.3 \| 3757724 \| \| wolf \| 454109.3 \| 3752388 \| \| wolf \| 398592.2 \| 3890488 \| \| wolf \| 419405.8 \| 3920082 \| \| wolf \| 404213.3 \| 3914210 \| \| wolf \| 430154.4 \| 3774276 \| \| wolf \| 427094.8 \| 3768638 \| \| wolf \| 416335 \| 3758352 \| \| wolf \| 416172.3 \| 3762053 \| \| wolf \| 418534.2 \| 3777047 \| \| wolf \| 374254.8 \| 3741535 \| \| wolf \| 413113.7 \| 3820405 \| \| wolf \| 403133.5 \| 3827509 \| \| wolf \| 386654.6 \| 3792603 \| \| wolf \| 455805.8 \| 3877118 \| \| wolf \| 455732.3 \| 3862511 \| \| wolf \| 486871.2 \| 3907291 \| \| wolf \| 481408.2 \| 3925532 \| \| wolf \| 471072.4 \| 3913232 \| \| wolf \| 450458.3 \| 3893495 \| |
| --- | --- | --- | --- | --- | --- | --- | --- | --- | --- | --- | --- | --- | --- | --- | --- | --- | --- | --- | --- | --- | --- | --- | --- | --- | --- | --- | --- | --- | --- | --- | --- | --- | --- | --- | --- | --- | --- | --- | --- | --- | --- | --- | --- | --- | --- | --- | --- | --- | --- | --- | --- | --- | --- | --- | --- | --- | --- | --- | --- | --- | --- | --- | --- | --- | --- | --- | --- | --- | --- | --- | --- | --- | --- | --- | --- | --- | --- | --- | --- | --- | --- | --- | --- | --- | --- | --- | --- | --- | --- | --- | --- | --- | --- | --- | --- | --- | --- | --- | --- | --- | --- | --- | --- | --- | --- | --- | --- | --- | --- | --- | --- | --- | --- | --- | --- | --- | --- | --- | --- | --- | --- | --- | --- | --- | --- | --- | --- | --- | --- | --- | --- | --- | --- | --- | --- | --- | --- | --- | --- | --- | --- | --- | --- | --- | --- | --- | --- | --- | --- | --- | --- | --- | --- | --- | --- | --- | --- | --- | --- | --- | --- | --- | --- | --- | --- | --- | --- | --- | --- | --- | --- | --- | --- | --- | --- | --- | --- | --- | --- | --- | --- | --- | --- | --- | --- | --- | --- | --- | --- | --- | --- | --- | --- | --- | --- | --- | --- | --- | --- | --- | --- | --- | --- | --- | --- | --- | --- | --- | --- | --- | --- | --- | --- | --- | --- | --- | --- | --- | --- | --- | --- | --- | --- | --- | --- | --- | --- | --- | --- | --- | --- | --- | --- | --- | --- | --- | --- | --- | --- | --- | --- | --- | --- | --- | --- | --- | --- | --- | --- | --- | --- | --- | --- | --- | --- | --- | --- | --- | --- | --- | --- | --- | --- | --- | --- | --- | --- | --- | --- | --- | --- | --- | --- | --- | --- | --- | --- | --- | --- | --- | --- | --- | --- | --- | --- | --- | --- | --- | --- | --- | --- |
