# Supplementary material for: Identifying connectivity for two sympatric carnivores in human-dominated landscapes in central Iran
Source: PLoS One. 2022 Jun 16;17(6):e0269179. doi: 10.1371/journal.pone.0269179 (PMC9202930; doi:10.1371/journal.pone.0269179)
Supplement: S2 Table — (DOCX) [file pone.0269179.s007.docx]

Table S2: Environmental and anthropogenic variables and their sources.

| **Variable**  **(Unit of measurement)** | **Description** | **Source of data** |
| --- | --- | --- |
| Elevation (m) | Digital Elevation Model (DEM) | http://earthexplorer.usgs.gov |
| Topographic roughness (m) | The amount of elevation difference between adjacent cells | Digital Elevation Model (DEM, http://earthexplorer.usgs.gov) |
| vegetation cover (Density of vegetation types (%)) | vegetation types with density higher than 25 % from the land cover/ land use map of the study area | Republished from [ http://www.frw.ir] under a CC BY license, with permission from [Forest, Range, Watershed Management Organization of Markazi province (IFRWO)], original copyright [2021]. |
| NDVI | Normalized Difference Vegetation Index | Landsat 8 OLI images (http://earthexplorer.usgs.gov/) |
| Distance to roads (m) | Euclidean distance to roads | https://data.humdata.org/dataset/wfp-geonode-iran-road-network-main-roads |
| Distance to human settlements (m) | Euclidean distance to villages | https://mapcruzin.com/free-iran-arcgis-maps-shapefiles.htm |
| Distance to dumpsites (m) | Euclidean distance to dumpsites | This data was gathered by the first author of this research |
| Distance to CAs (m) | Euclidean distance to Conservation Areas. | Republished from [ https://markazi.doe.ir/] under a CC BY license, with permission from [Markazi Province Office of Department of Environment (DOE)], original copyright [2021]. |
| Slope (%) | The rate of change of elevation for each cell | DEM (http://earthexplorer.usgs.gov) |
