# Supplementary material for: Identifying connectivity for two sympatric carnivores in human-dominated landscapes in central Iran
Source: PLoS One. 2022 Jun 16;17(6):e0269179. doi: 10.1371/journal.pone.0269179 (PMC9202930; doi:10.1371/journal.pone.0269179)
Supplement: S3 Table — Republished from [http://www.frw.ir] under a CC BY license, with permission from [Forest, Range, Watershed Management Organization of Markazi province (IFRWO)], original copyright [2021]. Republished from [https://markazi.doe.ir/] under a CC BY license, with permission from [Markazi Province Office of Department of Environment (DOE)], original copyright [2021]. (DOCX) [file pone.0269179.s008.docx]

Table S3- Variable contribution in the habitat modeling of the grey wolf and golden jackal in central Iran. Republished from [ [http://www.frw.ir](http://www.frw.ir/)] under a CC BY license, with permission from [Forest, Range, Watershed Management Organization of Markazi province (IFRWO)], original copyright [2021]. Republished from [ https://markazi.doe.ir/] under a CC BY license, with permission from [Markazi Province Office of Department of Environment (DOE)], original copyright [2021].

|  | | Elevation | Topographic roughness | Distance from CAs | NDVI | Distance from human settlements | Distance from dump sites | Distance from roads |
| --- | --- | --- | --- | --- | --- | --- | --- | --- |
| Grey wolf | Mean | **25** | **30** | **22** | 8 | 4 | **7** | 4 |
|  | SD | 5.8 | 1.2 | 2.2 | 4.6 | 1.4 | 1.8 | 0.8 |
| Golden jackal | Mean | 10 | **20** | **7** | 5 | **35** | 15 | 8 |
|  | SD | 2.2 | 3.6 | 1.9 | 1.5 | 1.6 | 2.3 | 0.9 |
